# Validation of rectal sparing throughout the course of proton therapy treatment in prostate cancer patients treated with SpaceOAR^®^

**DOI:** 10.1002/acm2.12010

**Published:** 2016-11-30

**Authors:** Samantha G. Hedrick, Marcio Fagundes, Sara Case, Jackson Renegar, Marc Blakey, Mark Artz, Hao Chen, Ben Robison, Niek Schreuder

**Affiliations:** ^1^ Provision Center for Proton Therapy Knoxville TN USA

**Keywords:** hydrogel, prostate, proton therapy, QACT, SpaceOAR

## Abstract

The purpose of this study was to investigate the consistency of rectal sparing using multiple periodic quality assurance computerized tomography imaging scans (QACT) obtained during the course of proton therapy for patients with prostate cancer treated with a hydrogel spacer. Forty‐one low‐ and intermediate‐risk prostate cancer patients treated with image‐guided proton therapy with rectal spacer hydrogel were analyzed. To assess the reproducibility of rectal sparing with the hydrogel spacer, three to four QACTs were performed for each patient on day 1 and during weeks 1, 3, and 5 of treatment. The treatment plan was calculated on the QACT and the rectum V90%, V75%, V65%, V50%, and V40% were evaluated. For the retrospective analysis, we evaluated each QACT and compared it to the corresponding treatment planning CT (TPCT), to determine the average change in rectum DVH points. We were also interested in how many patients exceeded an upper rectum V90% threshold on a QACT. Finally, we were interested in a correlation between rectum volume and V90%. On each QACT, if the rectum V90% exceeded the upper threshold of 6%, the attending physician was notified and the patient was typically prescribed additional stool softeners or laxatives and reminded of dietary compliance. In all cases of the rectum V90% exceeding the threshold, the patient had increased gas and/or stool, compared to the TPCT. On average, the rectum V90% calculated on the QACT was 0.81% higher than that calculated on the TPCT. The average increase in V75%, V65%, V50%, and V40% on the QACT was 1.38%, 1.59%, 1.87%, and 2.17%, respectively. The rectum V90% was within ± 1% of the treatment planning dose in 71.2% of the QACTs, and within ± 5% in 93.2% of the QACTs. The 6% threshold for rectum V90% was exceeded in 7 out of 144 QACTs (4.8%), identified in 5 of the 41 patients. We evaluated the average rectum V90% across all QACTs for each of these patients, and it was found that the rectum V90% never exceeded 6%. 53% of the QACTs had a rectum volume within 5 cm^3^ of the TPCT volume, 68% were within 10 cm^3^. We found that patients who exceeded the threshold on one or more QACTs had a lower TPCT rectal volume than the overall average. By extrapolating patient anatomy from three to four QACT scans, we have shown that the use of hydrogel in conjunction with our patient diet program and use of stool softeners is effective in achieving consistent rectal sparing in patients undergoing proton therapy.

## 1 Introduction

In the management of localized prostate cancer, multiple treatment options have been employed, including radical prostatectomy, external beam radiation therapy using intensity‐modulated photon beam (IMRT), brachytherapy, cryotherapy, and proton therapy.^1^, ^2^, ^3^ Proton therapy provides an advantage over IMRT by reducing the radiation dose delivered to normal tissues outside of the target volume.^4^, ^5^ Image‐guided treatment delivery, as well as use of endorectal balloons (ERB), have been historically employed to allow for tighter planning target margins thus decreasing the volume of rectum exposed to high radiation doses.^6^, ^7^, ^8^, ^9^ However, due to the proximity of the anterior rectal wall to the prostate, high radiation doses to the rectum are still delivered by the various radiotherapy techniques, including protons.

Endorectal balloons will naturally distend the rectum and result in the displacement of the anterior rectal wall into the prostate, thus into the high dose region. A novel concept consisting of the use of a tissue spacer to displace the anterior rectal wall away from the prostate has been shown to be feasible and to remain stable during the course of treatment, with the advantage of significantly decreasing rectal irradiation.^10^, ^11^, ^12^, ^13^, ^14^, ^15^ In a multicenter trial, high dose rectal sparing with hydrogel spacer for patients undergoing IMRT was associated with reduced rectal toxicity severity and improved bowel quality of life scores.^16^ The commercial availability of a polyethylene‐glycol hydrogel absorbable water spacer (SpaceOAR, Augmenix, Inc., Waltham, MA, USA) has led to increased interest in adopting this method to minimize radiation‐induced rectal toxicity.^17^ Our clinic has been utilizing SpaceOAR since its FDA approval in April 2015, and we have treated over 250 patients with the gel spacer and proton therapy, as of June 2016. We have treated with both uniform scanning (US) and pencil beam scanning (PBS), with either ERB or hydrogel spacer, and our results comparing the two treatment modalities are presented elsewhere.^18^, ^19^

The purpose of this study was to investigate the consistency of rectal sparing using multiple periodic quality assurance computerized tomography imaging (QACT) obtained during the course of proton therapy treatment. To our knowledge, this is the first study to analyze rectal dose sparing reproducibility in patients treated with proton therapy and rectal spacer.

## 2 Methods and materials

Forty‐one patients with low‐ or intermediate‐risk prostate cancer treated with image‐guided proton therapy with rectal spacer hydrogel between April 2015 and December 2015 were analyzed in this study.

### 2.A Hydrogel implant

All hydrogel and fiducial marker placement were performed during the same outpatient procedure under local perineum skin numbing, following general application technique guidelines previously published.^20^ Specifically, at our facility, all patients had a fleet enema 2–3 h prior to the procedure. The patient was placed in lithotomy position, and the perineum was prepped with chlorhexidine. A biplane linear side‐fire transrectal ultrasound (TRUS) probe was used for proper visualization of the anatomy and guidance of the needle placement. The needle was inserted into the subcutaneous tissue at midline, 1–1.5 cm anterior to the anal verge. Around 2–3 cm^3^ of anesthetic (2% lidocaine without vasoconstrictor buffered with bicarbonate 8.4%, mixed 10:1) was injected just beneath the skin. The needle was then carefully advanced under ultrasound visualization to abut the prostate apex just to the right and left of midline, where an additional 2–3 cm^3^ of anesthetic was injected in each side. For each patient, before inserting the gel, three fiducial markers were implanted: one in the right posterior base, one in the right posterior apex, and one placed in the left anterior midgland. The SpaceOAR needle was attached to a 10‐cm^3^ saline syringe and then inserted 1 cm above the TRUS at midline, and it was advanced parallel to the ultrasound probe to penetrate the rectoprostatic space, just beneath the Denonvillier's fascia staying outside of the rectal wall. A single radiation oncologist performed all hydrogel and fiducial placements. There were no instances of rectal needle penetration and all procedures were performed without complications.

### 2.B Planning and treatment

Each patient underwent a treatment planning CT (TPCT) scan approximately 1‐week post implant. On the same day, patients also received several MRI scans to visualize the hydrogel. At the time of simulation, patients were instructed to administer a fleet enema 2–3 h prior to the CT. Fleet enemas were not repeated for each treatment day, unless necessary. Both simulation CT and MRI scans were performed sequentially on the same day with an empty rectum, to provide the best‐case scenario. Patients were also instructed to have a nearly full bladder for treatment, and the drinking water volume and time‐prior‐to‐treatment was recorded by therapists to replicate for each treatment. Patients were instructed to maintain a low residue diet and use a daily gas prevention medicine.

**Figure 1:**
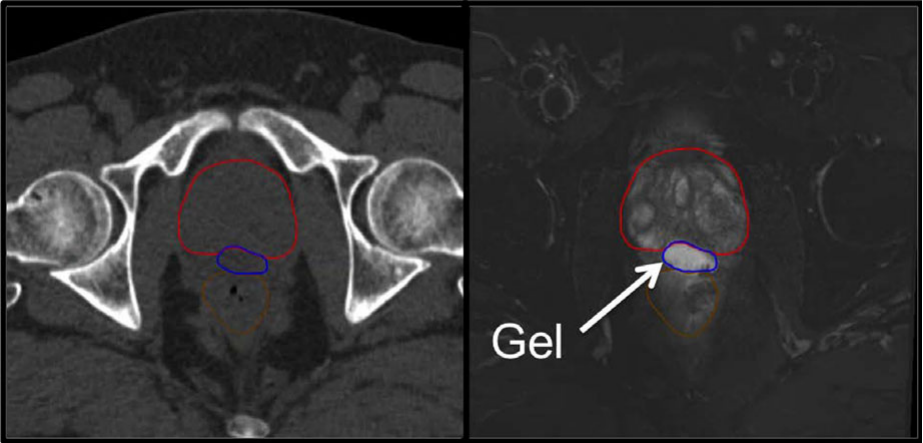
Left: treatment planning CT (TPCT), indicating the prostate (red), hydrogel (blue), and rectum (brown). Right: MRI acquired on the same day to visualize the hydrogel.

The MRIs were fused to the TPCT based on three implanted gold fiducials in the prostate. Figure 1 demonstrates the registration between the TPCT and MRI and the ability to visualize the gel on the MRI. The prostate, seminal vesicles, gel, and rectum were contoured on the MRI and transferred to the TPCT. Planning was performed on the TPCT.

The clinical target volume (CTV) for low‐risk patients included only the prostate, as visualized on MRI and CT fusion; the CTV for intermediate risk included the proximal and medial 1 cm of the seminal vesicles on the first phase of the treatment with a subsequent boost to CTV2, defined as the prostate. The planning target volume (PTV) is an expansion of the CTV, 5 mm posteriorly and 6 mm elsewhere. In addition, the PTV Evaluation (PTV_Eval) structure is an expansion of the PTV, 5 mm laterally. PTV_Eval was used for inverse planning to increase dose range laterally, which improves dose coverage and overall plan robustness.

Based on eligibility and patient preference, low‐ and intermediate‐risk patients were assigned to either conventional fractionation (27 patients) or hypofractionation (14 patients) prescriptions. Conventional fractionation patients received 78 Gy_(RBE)_ in 39 fractions, with intermediate‐risk patients receiving 52 Gy_(RBE)_ to the prostate and proximal seminal vesicles and 26 Gy_(RBE)_ to the prostate only. Hypofractionation patients received 62 Gy_(RBE)_ in 20 fractions, with intermediate‐risk patients receiving 40.3 Gy_(RBE)_ to the prostate and proximal seminal vesicles and 21.7 Gy_(RBE)_ to the prostate only.

Treatment was planned using the RayStation planning system with the following objectives and dose constraints: For target coverage, 95% of the PTV was to receive 100% of the prescribed dose and 100% of the PTV was planned to receive a minimum of 95% of the prescription dose. We have previously documented the ability to reach a rectum V90% of ≤ 1% using pencil beam scanning proton therapy and SpaceOAR with the margins and expansions described above, so our rectum OAR constraint was routinely set at V90% ≤ 1% while maintaining target coverage priority.^18^, ^19^ Each patient in this study was treated with two opposing lateral fields. For each plan, the rectum V90%, V75%, V65%, V50%, and V40% were evaluated. Each patient underwent a robust evaluation by a medical physicist, analyzing the effects of 5 mm of motion in all directions, 3 degrees of roll, 3 degrees of yaw, and 2.5% + 1 mm range uncertainty. Under all perturbations, the prostate CTV must maintain V100% ≥ 95%.

In the treatment room, patients were imaged with orthogonal x‐rays and aligned based on fiducials. Each fiducial contour on the DRR had a 2‐mm uniform expansion. Patients were imaged before each field and the table was translated, as necessary, to align the fiducials within the expanded contours.

### 2.C QACT analysis

To assess the reproducibility of rectal sparing with the hydrogel spacer, three to four quality assurance CTs (QACT) were performed for each patient on day 1 and during weeks 1, 3, and 5 of treatment. Each patient was set up in the treatment position and scanned. These QACTs were performed either immediately before or after the patient's treatment, so bladder filling was not necessarily at the ideal volume for treatment. A physicist would then fuse the QACT to the TPCT, based on fiducials, and deform the contours to the QACT. A physicist would analyze the deformed contours and make changes, as necessary. The treatment plan was then calculated on the QACT and the rectum V90%, V75%, V65%, V50%, and V40% were evaluated. Because the use of hydrogel spacers has been shown to significantly improve rectum V90%, we chose to use V90% as our prospective action point.^16^

**Figure 2:**
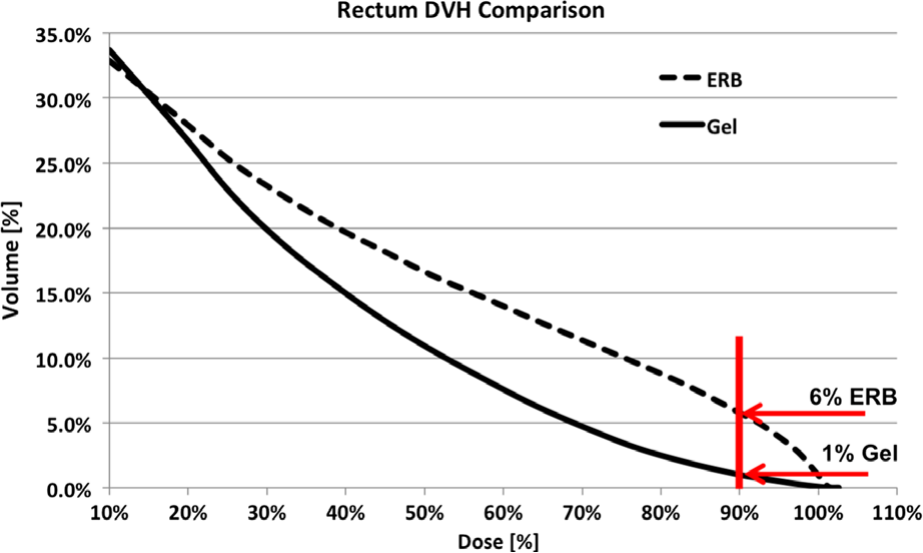
DVH comparison of our average ERB data (dashed) versus our average gel data (solid).

In a previous study, we evaluated the improvement in rectal sparing with gel compared to an ERB.^19^ Based on our previous data, our ERB average rectum V90% is 6%. In this study, the gel was considered to have reproducibly spared the rectum on a QACT when it improved upon the ERB values. Figure 2 illustrates the average rectum DVH values for our ERB and gel patients. We used this 6% value as an upper threshold limit when analyzing QACTs.

The analysis of each QACT was performed within 1 day of CT acquisition, and the results of each analysis were reported to the attending physician. If the rectum V90% on a QACT exceeded our threshold, actions were taken, as discussed in the results section.

Retrospective analysis was also performed on each QACT, evaluating the rectum DVH points. For the retrospective analysis, we evaluated each QACT and compared it to the corresponding TPCT to determine the average change in rectum DVH points. We were also interested in how many patients exceeded the upper rectum V90% threshold on a QACT. Finally, we were interested in a correlation between rectum volume and V90%. PTV coverage and bladder sparing were also evaluated for consistency, but were not part of this study.

For this study, we did not analyze the hydrogel separation on each QACT. A detailed analysis of hydrogel separation throughout treatment would require sequential MRI scans, which is not typically feasible due to insurance coverage and limitations. Song et al. found that the hydrogel volume is stable throughout the course of treatment.^13^ Therefore, we would not expect to identify a change in gel separation over time, up to 3 months.

## 3 Results

All patients tolerated fiducial and gel implants well and successfully completed treatment. A total of 144 QACT scans were analyzed, with each patient receiving three to four QACTs. Data of the analyzed patient population are in (Table 1). Data include the time between the gel implant and the TPCT, time between the gel implant and the first fraction, prostate volume, as measured on MRI, and gel separation distance between the prostate and rectum, measured at the centroid of the prostate. The data are included for the average of all 41 patients and separated into the patients who had at least one QACT that exceeded the rectum V90% upper threshold and those who were consistently below the threshold.

**Table 1:** Patient population

	All patients	Patients below threshold	Patients above threshold
Number of patients	41	36	5
Avg gel‐to‐TPCT time (d)	4 (±1)	4 (±1)	4 (±1)
Avg gel‐to‐1st Fx time (d)	19 (±4)	19 (±4)	20 (±6)
Avg prostate volume (cm^3^)	62.6 (±21.7)	62.02 (±22.8)	66.78 (±12.1)
Avg gel separation (cm)	1.34 (±0.21)	1.31 (±0.16)	1.54 (±0.38)

Patient population data are included for the average of all 41 patients and separated in to those patients who had at least one QACT that exceeded the rectum V90% threshold of 6% (“Patients above threshold”) and those that did not (“Patients below threshold”). Average values are reported with one standard deviation.

### 3.A QACT analysis during treatment

**Figure 3:**
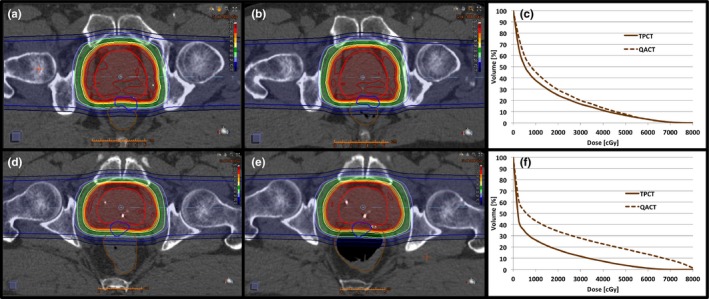
Top: example of a QACT below the threshold. (a) TPCT, (b) QACT, (c) rectum DVH comparison. TPCT (solid), QACT (dotted). Bottom: example of a QACT that exceeded the threshold. (d) TPCT, (e) QACT, (f) rectum DVH comparison. TPCT (solid), QACT (dotted).

On each QACT, if the rectum V90% exceeded the upper threshold of 6%, the attending physician was notified and the patient was typically prescribed additional stool softeners or laxatives and reminded of dietary compliance. In all cases of the rectum V90% exceeding the threshold, the patient had increased gas and/or stool, compared to the TPCT. This led to a portion of the rectum deforming around the hydrogel and protruding into the prescription dose cloud**.** Figure 3 demonstrates results for a QACT analysis that remained below the threshold and a QACT that exceeded the threshold. The QACT in the bottom portion of the figure demonstrates the event when increased rectal filling distends the rectum around the gel. The DVH to the right shows the effect this increased filling has on the QACT analysis (dotted), compared to the TPCT (solid). For most patients, after additional measures were taken, the following QACT showed improved rectal sparing and passed our criteria. A majority of the patients did not exceed this threshold on any QACT, so no additional measures were taken.

### 3.B Retrospective QACT analysis

The QACT rectum DVH points for each patient were compared to their respective TPCT DVH points. It was assumed that the entire treatment dose was delivered to each of the QACTs, to simulate a worst‐case scenario. The rectum V90% calculated on a QACT was, on average, 0.81% (± 2.04%) higher than that calculated on the TPCT, one standard deviation included in parentheses. For example, a patient with a TPCT rectum V90% value of 1% had a QACT rectum V90% value of 1.81%. The data for rectum V90%, V75%, V65%, V50%, and V40% are shown in (Table 2). For each of the DVH points, the average volume change on the QACTs is positive, that is, the QACT value is higher than the TPCT.

**Table 2:** Dose–volume comparison of QACT vs TPCT

Rectum DVH	Average change from TPCT	Within 1%	Within 5%
V90%	0.81% (±2.04%)	71.2%	93.2%
V75%	1.38% (±3.26%)	49.3%	86.3%
V65%	1.59% (±4.34%)	34.2%	78.8%
V50%	1.87% (±5.13%)	26.0%	71.2%
V40%	2.17% (±8.55%)	19.2%	63.0%

**Figure 4:**
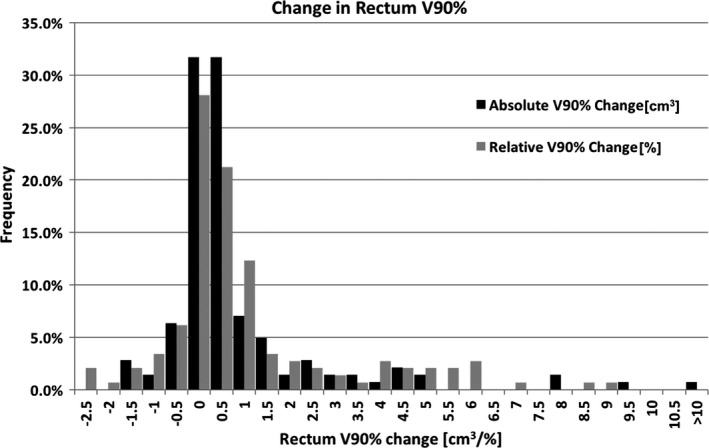
Distribution of average and relative change between each QACT and its respective TPCT. Positive change represents a higher QACT DVH value. Black bars are the absolute change in rectum V90%, in cm^3^. Grey bars are the relative change in rectum V90%, in percent.

The rectum V90% was within ± 1% of the treatment planning dose in 71.2% of the QACTs, and within ± 5% in 93.2% of the QACTs. Again, this 1% change is, for example, a rectum V90% increase from 1% on the TPCT to 2% on a QACT. For this analysis, each QACT was treated as an individual data point, rather than grouping them by patient. This is visualized in Fig. 4, where a positive change on the histogram represents a higher QACT V90%, compared to the TPCT. We were also interested in the difference when analyzing change in absolute volume of the rectum receiving 90% and relative volume receiving 90%. The relative change is the data reported above, that is, the change in percent volume receiving 90% of the prescription dose. These data are shown in the grey bars in the histogram. We also analyzed the change in rectum V90% absolute volume, that is, the change in cubic centimeters, rather than percent of rectum volume. This is shown in the black bars in the histogram. Because the rectum V90% relative volume change is dependent on the rectum volume on both the TPCT and the QACT, it may not correlate with the absolute amount of rectum receiving 90% of the prescription dose. There were occasions of increased rectal volume leading to posterior expansion, typically not increasing the V90%, or leading to anterior expansion around the gel, which would lead to increased V90%. To eliminate this variation, we analyzed the change in absolute volume of the rectum receiving 90%. Based on these results, we found that the absolute volume change (cm^3^) between the QACT and TPCT was less than the relative change (%), on average. Around 78% of QACTs had a rectum V90% within 1 cm^3^ of the TPCT, and 97% were within 5 cm^3^.

We also evaluated the change in rectal volume and found that 53% of the QACTs had a rectum absolute volume within 5 cm^3^ of the TPCT volume, 68% were within 10 cm^3^.

The 6% threshold for rectum V90% was exceeded in 7 out of 144 QACT's (4.8%), identified in 5 of the 41 patients. These five patients were further investigated, specifically looking at all of the QACTs for each patient. The TPCT and each QACT rectum V90% for these five patients are presented in (Table 3). The QACTs with rectum V90% that exceeded the 6% threshold are highlighted. Four of the five patients had only one QACT that exceeded the threshold, while the 5th patient had three of the four QACTs exceed the threshold. We evaluated the average rectum V90% for each of these five patients and found that the average rectum V90% never exceeded 6%. The QACTs that exceeded the 6% threshold could rather be seen as outliers or could be correlated with a situation where rectum filling was not necessarily representative of the entire treatment.

**Table 3:** Data for five patients with at least one QACT that exceeded the rectum V90% threshold

	TPCT [%]	QACT1 [%]	QACT2 [%]	QACT3 [%]	QACT4 [%]	QACT Avg [%]
Patient 1	2.68	2.85	8.45	1.96	N/A	4.42
Patient 2	0.23	7.83	3.75	1.30	3.53	4.10
Patient 3	0.02	8.44	0.30	0.10	0.22	2.27
Patient 4	4.59	4.32	5.34	1.69	8.01	4.84
Patient 5	1.70	7.27	6.99	1.39	6.32	5.49

TPCT and QACT rectum V90% for the five patients who had at least one QACT that exceeded the rectum V90% threshold of 6% (highlighted QACTs).

It is interesting to note that the average rectum volume on the TPCT for the five patients, where at least one QACT exceeded the 6% threshold, was 50.6 cm^3^ (± 10.0 cm^3^) compared to 62.1 cm^3^ (± 19.2 cm^3^) on the remaining patients (36 patients). Additionally, the average rectum V90% on the TPCT for these five patients was 2.1% (± 2.0%), compared to 0.8% (± 1.1%) on the remaining 36 patients. The higher TPCT rectum V90% appears to be a function of a smaller rectum, rather than a factor of the gel separation. In fact, the average gel separation was 1.54 cm (± 0.38 cm) for the five patients above the threshold and 1.31 cm (± 0.16 cm) for the remaining patients.

## 4 Discussion

The purpose of this study was to evaluate the reproducibility of high dose rectal sparing throughout treatment, based on several QACTs**,** in patients treated with image‐guided proton therapy with SpaceOAR hydrogel. The QACT scans were compared to the nominal plan obtained in patients with an empty rectum, that is, best‐case scenario. Some rectal filling with stools, gas, or both would necessarily have to be expected during treatment, thus potentially resulting in a degree of rectal distension and expansion of the anterior rectal wall toward the prostate gland, thus into a higher dose region. Furthermore, our analysis only considered three to four time points in each patient's treatment, so a single QACT is not necessarily representative of the entire treatment. Yet another limitation resides in the fact that QACTs were obtained off line and not temporally coincident with treatment delivery.

Nevertheless, even without a daily enema to reproduce the planning CT empty rectum, 71% of our hydrogel spacer patients had a rectal V90% that did not vary more than 1% when compared to the TPCT. Rectal V90% was within 5% of planned dose in just over 93% of instances.

Increased rectal filling caused by excessive stools, gas, or both was routinely identified as the culprit for increased rectal dose. Following planning CT/MRI to QACT image fusion and recontouring verification, no changes in gel thickness were identified as a confounding factor. We therefore chose a rectum V90% > 6% to prompt further intensification of stool softeners, use of laxatives, and addition of antigas medication to improve rectal emptying.

The choice of the rectum V90% > 6% threshold was based on our previous experience with ERB, where the mean rectum volume receiving 90% of prescribed dose was above 6%. It should be noted that the typical total rectum volume in patients treated with ERB at our center is 145 cm^3^. In such a case, for example, the absolute rectum volume exposed to 90% of the prescribed dose would be 8.7 cm^3^. By comparison, the mean total rectal volume in this population was 60 cm^3^, in which case 6% would only represent 3.6 cm^3^ of the rectum exposed to the same nominal dose. Therefore, we felt comfortable accepting such a threshold, prior to prompting additional repeat QACTs for confirmation of improved rectum emptying and thus dose sparing.

Even when we evaluated the absolute QACT rectum volume, which was increased, the mean was 64 cm^3^, in which case 6% would only represent 3.8 cm^3^. Based on our results of the absolute change in rectum V90%, we found that the absolute change was, on average, less than the relative change. This negated the effect of rectal filling on the DVH and only looked at the effect of rectal filling on change in absolute volume receiving 90% of the prescription.

When evaluating the seven QACTs that exceeded the rectum V90% threshold, the maximum absolute rectum volume receiving 90% of the dose was 9.4 cm^3^, identified for patient 1. This one event is the only QACT to exceed the ERB average absolute rectum volume of 8.7 cm^3^. The average absolute rectum volume on these seven QACTs is 6.2 cm^3^. Even though the relative rectum volume receiving 90% of the dose for these seven QACTs exceeds the ERB average, the absolute rectal volume does not.

It is also worth noting that daily kV/kV orthogonal imaging for fiducial alignment did allow the ability to visualize the presence of gas and even increased presence of stools, in many cases, prompting the therapist to either evacuate the gas with a thin rectal tube or to have the patient visit the toilet for a bowel movement before treatment delivery. Because a combination of all these measures were taken, it is unlikely that a patient would have received multiple fractions with unusually large rectal filling.

The use of QACTs to evaluate the stability of the rectum geometry/filling has been instrumental in fine‐tuning our prostate gel workflow. Since the beginning of the gel program, we have introduced a daily gas prevention medicine and it has decreased the QACT failure rate. Additionally, we have identified patients who are likely not good candidates for the hydrogel implant, such as those with hip prostheses or chronic constipation. Hip prostheses, for proton therapy, often require a beam passing through the rectum, which is sensitive to changes in filling. For these patients, a balloon provides more consistent filling.

## 5 Conclusion

The efficacy of a SpaceOAR hydrogel in rectal sparing has been demonstrated in several dosimetric and clinical studies. Multiple authors had previously documented significant rectal sparing on a single planning CT, however, information was lacking regarding the stability of rectal sparing throughout treatment. The purpose of our study was to obtain this valuable information to evaluate and confirm the efficacy of this method across the duration of a treatment course. By extrapolating patient anatomy from three to four QACT scans, we have shown that the use of hydrogel in conjunction with our patient diet program and use of stool softeners is effective in achieving consistent rectal sparing in patients undergoing proton therapy.
